# Global health at a crossroads: training the next generation of global health practitioners for a post-aid era

**DOI:** 10.7189/jogh.15.02002

**Published:** 2025-11-14

**Authors:** Fan Wu, Jinkou Zhao

**Affiliations:** 1 Shanghai Medical College, Fudan University, Shanghai, China; 2 Fudan Institute for Advanced Studies in Global Health, Fudan University, Shanghai, China; 3 Shanghai Institute of Infectious Diseases and Biosecurity, Fudan University, Shanghai, China; 4 The Global Fund to Fight AIDS, Tuberculosis and Malaria, Geneve, Switzerland

From 23 June to 11 July 2025, the Fudan University Shanghai Institute of Infectious Diseases and Biosecurity and the Fudan Institute for Advanced Studies in Global Health successfully hosted the Fudan University Global Health Summer School. This flagship programme brought together master’s students and PhD candidates from across the globe to explore the interconnected systems, governance, and determinants that shape global health. It was structured around four interlinked modules, each addressing key thematic areas in global health theory and practice.

The opening module introduced foundational concepts of global health, covering transitions in epidemiology, demography, socioeconomic determinants, and behavioral and metabolic factors through a life course approach to health; what we learned from global response to COVID-19 pandemics and implications for pandemic preparedness; multi-disease elimination, with neglected tropical diseases as the example; universal health coverage and primary health care through lens of vaccination programmes. The first module also covered migrants and health; challenges and opportunities in refugee health inclusion; climate change, vaccines as tools of adaptation and mitigation; and the power of possibilities for global health in the era of artificial intelligence.

The second module provided a comprehensive overview of global health financing, tracing the historical evolution of funding models and highlighting current shifts as traditional donors retreat from official development assistance. It explored global health spending by income levels; where and how external aid, domestic financing, and out-of-pocket expenses played a role; main factors considered in the global health initiative investment cases through real examples of the Global Fund over different rounds; and innovative financing mechanisms such the International Finance Facility for Immunization, air ticket levies, debt to health swap, and blended financing, with Gavi, the Vaccine Alliance, the Global Fund, and Unitaid serving as examples. The second module also discussed how all sources of health financing can be optimised in-country to achieve universal health coverage and equity.

The third module explored the evolving global health governance and diplomacy amid growing geopolitical instability and the withdrawal of traditional public donors. It covered power reconstruction in the era when major donors are retreating from the official development assistance; global health players and stakeholders, agenda setting bodies, and decision-making platforms; state and non-state actors in the decision-making process using the examples from major global health initiatives (*e.g.* the Global Fund, Gavi, the Vaccine Alliance, Stop TB Partnership, Rollback Malaria, Unitaid, and the Pandemic Fund); global health diplomacy practices, the relationship between foreign policy and global health, using health attachés as the example to describe necessary competencies required for global health diplomats; and lessons learned from the intergovernmental negotiations for a pandemic agreement, the largest scale global health diplomacy. The potential of global health cooperation as a form of soft power was also discussed.

The final module covered global health in practices, including local production of health products, performance-based funding and accountability, and applications of innovative technologies to improve the access and use of services. Real examples from the Global Fund, Gavi, the Vaccine Alliance, Unitaid, were used in the training. This module also discussed about how the global level guidance, policy, and innovations are translated into the locally tailored practices to achieve universal health coverage and equity in the service delivery.

The Summer School invited 20 internationally renowned subject experts from leading global health institutions, academic institutions, universities, and think tanks to address the different transdisciplinary and interdisciplinary aspects of global health. These individuals brought cutting-edge knowledge and real-world insights, having been actively engaged in global-level discussions and decision-making processes. Presentations were followed by in-depth, student-led discussions guided by faculty facilitators from the Fudan University, using provocative questions posed by invited subject experts to stimulate debate and critical thinking.

Students were master’s students and PhD candidates from a wide range of disciplines, including public health, medicine, international relations, health economics, and political science, representing both the Fudan University and universities all over the world. International students came from various universities, such as the Harvard University, King’s College London, University of Geneva, Swiss Tropical and Public Health Institute, Mahidol University, University of Paris, among many others. Geographically, they represented Africa, Latin and North America, Asia, and Europe.

To facilitate interdisciplinary exchange, the students were divided into eight diverse groups, with each assigned a multidisciplinary subject topic prepared by invited subject experts. Under their guidance, students were tasked with exploring and debating respective subject topic through literature reviews, structured discussions, and manuscript drafting which are intended to be submitted to peer-reviewed journals.

Discussions among students and with invited subject experts were vibrant and often heated, covering pressing debates in global health, such as the implementation of vertical disease-specific programmes *vs.* integrated service delivery; the role of civil society and communities in global health governance, especially when members may face stigma or criminalisation; and how to align financing for universal health coverage amid shifting national and global priorities, among many others. These dialogues underscored the interconnectedness and interdependencies among health programmes, determinants, governance, financing, and diplomacy, highlighting the multidisciplinary and transdisciplinary nature of global health challenges and solutions.

As global health faces uncertainty, marked by the USA’s withdrawal from the World Health Organization and general cuts to funding due to retreats from traditional donors, the discipline may be entering a prolonged twilight – but it has not yet reached midnight. Equipping the next generation of global health practitioners with both historical perspective and up-to-date practical knowledge relevant for global health is an urgent imperative. These future global health players, whether consciously or not, will shape the discipline’s next chapter. With their insight, commitment, and collaboration, they may well light the way toward a new dawn.

